# Supplementary material for: ARID1B maintains mesenchymal stem cell quiescence via inhibition of BCL11B-mediated non-canonical Activin signaling
Source: Nat Commun. 2024 May 30;15:4614. doi: 10.1038/s41467-024-48285-2 (PMC11139927; doi:10.1038/s41467-024-48285-2)
Supplement: Supplementary file 1 — Supplementary Information [file 41467_2024_48285_MOESM1_ESM.pdf]

ARID1B maintains mesenchymal stem cell quiescence via inhibition of BCL11B-mediated non-canonical Activin signaling

This document includes:

Supplementary Fig. 1 to Supplementary Fig. 11

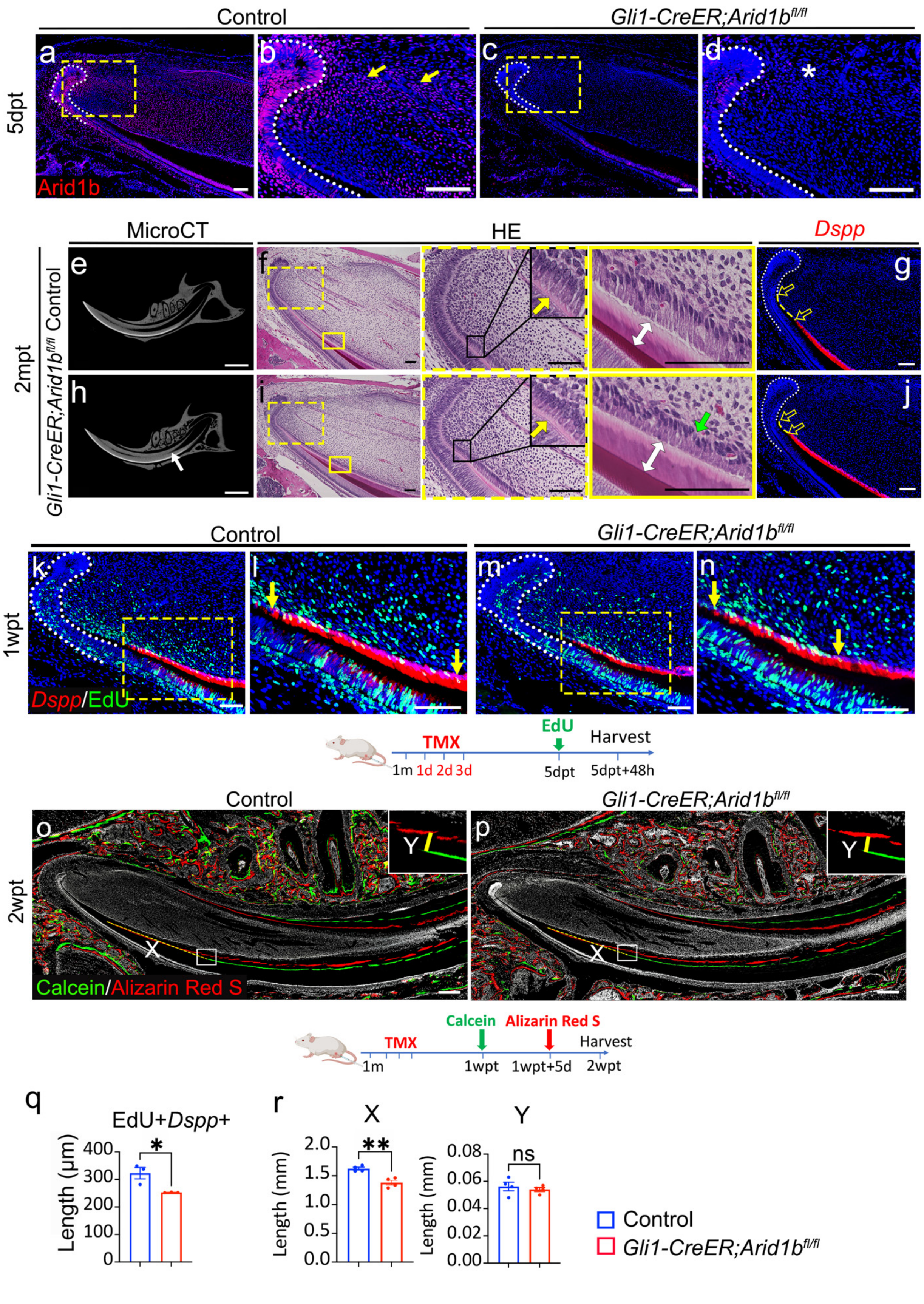
Control
Gli1-CreER;Arid1b^fl/fl
5dpt
Arid1b
MicroCT
HE
Dspp
2mpt
Control
Gli1-CreER;Arid1b^fl/fl
1wpt
Dspp/EdU
TMX
EdU
Harvest
1m 1d 2d 3d
5dpt
5dpt+48h
2wpt
Calcein/Alizarin Red S
Calcein
Alizarin Red S
1wpt
1wpt+5d
2wpt
EdU+Dspp+
Length (μm)
Length (mm)
X
Y
ns
Control
Gli1-CreER;Arid1b^fl/fl

Supplementary Figure 1 Loss of *Arid1b* impairs odontoblast migration in *Gli1-CreER;Arid1b$^{fl/fl}$* mouse incisor.

(a-d) ARID1B immunostaining in control and *Gli1-CreER;Arid1b$^{fl/fl}$* mouse incisors. b and d represent high-magnification image of the boxes in a and c. White dotted lines outline the cervical loop. Yellow arrows point to positive signals. Asterisk denotes the absence of target signal. dpt, day post-tamoxifen injection. n=3.

(e-j) MicroCT, HE staining, and *Dspp in situ* hybridization in control and *Gli1-CreER;Arid1b$^{fl/fl}$* mouse incisors. (e, h) MicroCT of control (e) and *Gli1-CreER;Arid1b$^{fl/fl}$* (h) mouse incisors. White arrow indicates the narrowed dental pulp. (f, i) HE staining of control (f) and *Gli1-CreER;Arid1b$^{fl/fl}$* (i) mouse incisors. Yellow arrows indicate the initiation of odontoblast polarization. Green arrows indicate the disordered alignment of odontoblasts. White two-way arrows indicate the dentin thickness. Boxes in f and i are shown at higher magnification on the right. (g, j) *Dspp* (red) *in situ* hybridization in control (g) and *Gli1-CreER;Arid1b$^{fl/fl}$* (j) mouse incisors. Yellow dotted lines show the cervical loop bending point to the initiation of odontoblast. Unfilled arrows indicate the distance between the yellow dotted lines. n=3.

(k-n) *Dspp* (red) *in situ* hybridization and EdU staining (green) in control (k, l) and *Gli1-CreER;Arid1b$^{fl/fl}$* (m, n) mouse incisor. l and n represent high-magnification images of boxes in k and m. Yellow arrows in l and n indicate the migration length of differentiated TACs. wpt, week post-tamoxifen injection.

(o-p) Double labeling of calcein and Alizarin red S in control (o) and *Gli1-CreER;Arid1b$^{fl/fl}$* (p) mouse incisor at 2 weeks after induction. X indicates the odontoblast migration length; Y indicates the dentin deposition depth.

(q) Quantification of EdU-labeled Dspp+ cells distance from control and *Gli1-CreER;Arid1b$^{fl/fl}$* mouse incisors. Data are mean±SEM, n=3, unpaired two-tailed Student's t-test, p=0.028.

(r) Quantification of the length of X and Y from control and *Gli1-CreER;Arid1b$^{fl/fl}$* mouse incisors. Data are mean±SEM, n=4, unpaired two-tailed Student's t-test, p=0.0023, ns, not significant.

Scale bars, 100 μm (a-d, f-g, i-n); 2 mm (e, h); 500 μm (o, p). The schematics under k-n and o-p created with BioRender.com released under a Creative Commons Attribution-NonCommercial-NoDerivs 4.0 International license.

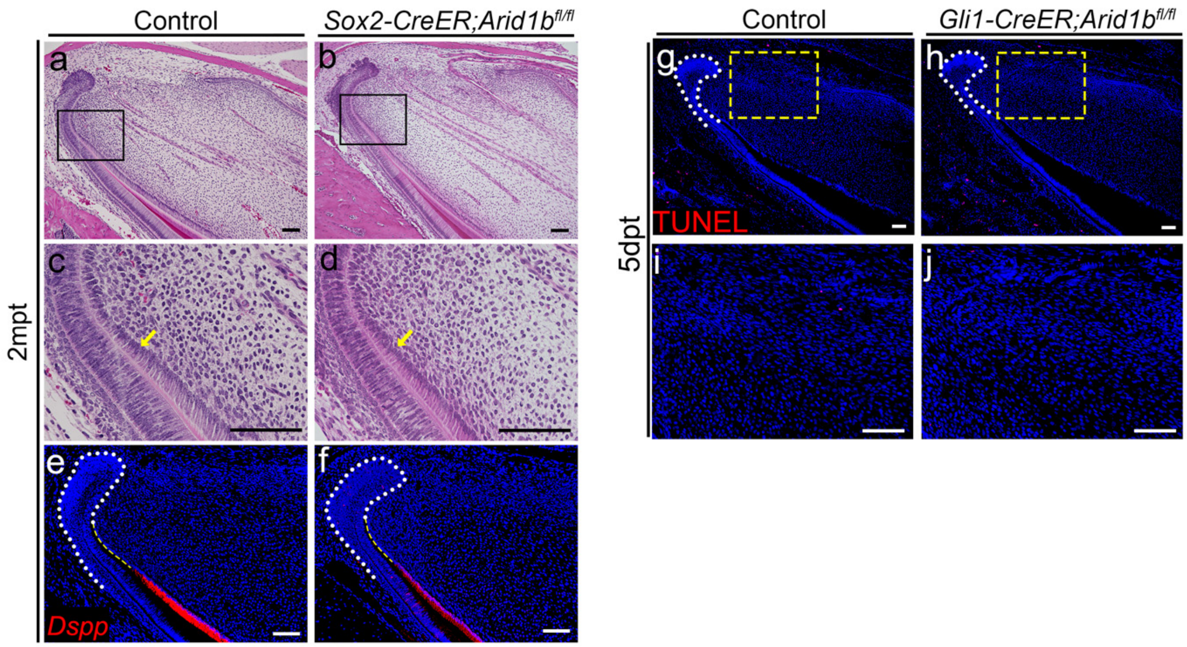

Supplementary Figure 2 Loss of *Arid1b* in the dental epithelium does not cause obvious dental mesenchymal defects.

(a-d) HE staining of control (a, c) and *Sox2-CreER;Arid1b*$^{fl/fl}$ (b, d) mouse incisors 2 months after tamoxifen injection. c and d represent high-magnification images of the boxes in a and b, respectively. Yellow arrows indicate the initiation of odontoblast polarization.

(e-f) *In situ* hybridization of *Dspp* in incisors of control (e) and *Sox2-CreER;Arid1b*$^{fl/fl}$ (f) mice 2 months after tamoxifen injection. White dotted lines outline the labial cervical loop. Yellow dotted lines show the distance between the bending point of the cervical loop and the initiation of odontoblast differentiation.

(g-j) TUNEL assay in the proximal region of the incisors from control (g, i) and *Gli1-CreER;Arid1b*$^{fl/fl}$ (h, j) mice at 5 days post-tamoxifen induction. i and j represent high-

magnification images of the boxes in g and h, respectively. White dotted lines outline the labial cervical loop.

All the images are representative of at least 3 biological replicates. Scale bars, 100 μm.

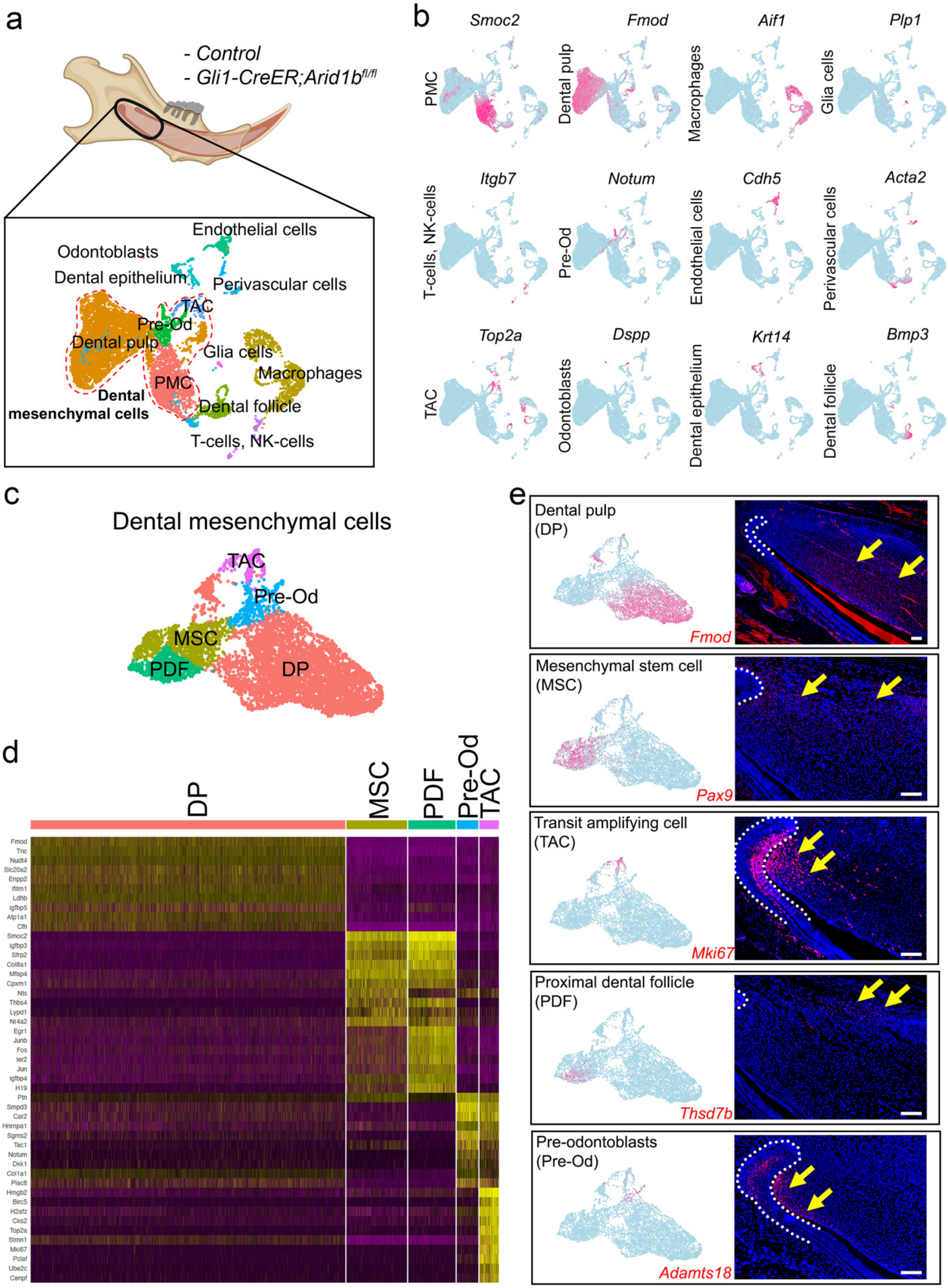
a
- Control
- Gli1-CreER;Arid1b fl/fl
Endothelial cells
Odontoblasts
Dental epithelium
Perivascular cells
TAC
Pre-Od
Dental pulp
Glia cells
Macrophages
PMC
Dental mesenchymal cells
Dental follicle
T-cells, NK-cells
b
Smoc2
Fmod
Aif1
Plp1
PMC
Dental pulp
Macrophages
Glia cells
Itgb7
Notum
Cdh5
Acta2
T-cells, NK-cells
Pre-Od
Endothelial cells
Perivascular cells
Top2a
Dspp
Krt14
Bmp3
TAC
Odontoblasts
Dental epithelium
Dental follicle
c
Dental mesenchymal cells
TAC
Pre-Od
MSC
PDF
DP
d
DP
MSC
PDF
Pre-Od
TAC
e
Dental pulp (DP)
Fmod
Mesenchymal stem cell (MSC)
Pax9
Transit amplifying cell (TAC)
Mki67
Proximal dental follicle (PDF)
Thsd7b
Pre-odontoblasts (Pre-Od)
Adamts18

Supplementary Figure 3 scRNA-seq cluster profiles of control and *Gli1-CreER;Arid1b$^{fl/fl}$* mouse incisor samples.

(a-b) UMAP plot of all cell types in control and *Gli1-CreER;Arid1b$^{fl/fl}$* mouse incisor samples, and FeaturePlot of marker genes in the mouse incisor and its surrounding tissue. Schematic drawing of the mouse incisor created with BioRender.com released under a Creative Commons Attribution-NonCommercial-NoDerivs 4.0 International license. Cell types were annotated based on the expression of cell type-specific marker genes. PMC, proximal mesenchymal cell; TAC, transit-amplifying cell; Pre-Od, pre-odontoblast.

(c) UMAP plot of annotated dental mesenchymal cell types in control and *Gli1-CreER;Arid1b$^{fl/fl}$* mouse incisor samples. MSC, mesenchymal stem cell; PDF, proximal dental follicle; TAC, transit amplifying cell; DP, dental pulp; Pre-Od, pre-odontoblast.

(d) Heatmap of signature genes in the dental mesenchymal cell clusters of the mouse incisor.

(e) FeaturePlot of marker genes and their corresponding *in situ* hybridization in the mouse incisor dental mesenchymal cells.

All the immunostaining *in situ* hybridization images represent at least 3 biological replicates. Scale bars: 100 µm.

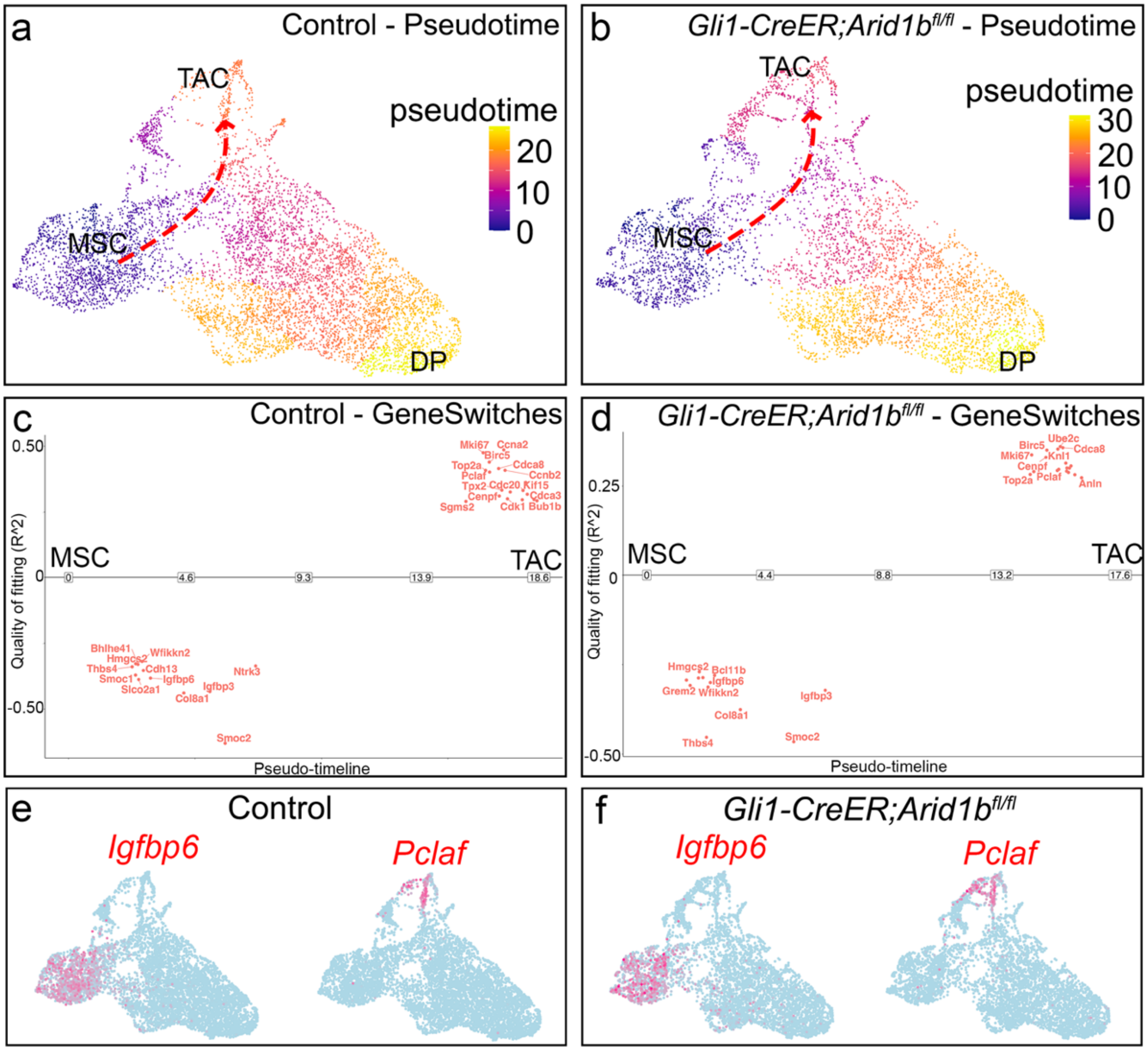

Supplementary Figure 4 GeneSwitches analysis in MSC to TAC trajectory between control and *Gli1-CreER;Arid1b*$^{fl/fl}$ mouse incisor samples.

(a-b) Pseudotime analysis for dental mesenchymal cells from control (a) and *Gli1-CreER;Arid1b*$^{fl/fl}$ (b) mouse incisor samples. MSC, mesenchymal stem cell; TAC, transit-amplifying cell; DP, dental pulp.

(c-d) GeneSwitches analysis of the MSC to TAC trajectory in control (c) and *Gli1-CreER;Arid1b*$^{fl/fl}$ (d) mouse incisor samples.

(e-f) FeaturePlot of cluster-specific activated genes identified from GeneSwitches analysis and gene expression level comparison between control (e) and *Gli1-CreER;Arid1b*$^{fl/fl}$ (f) mouse incisor samples.

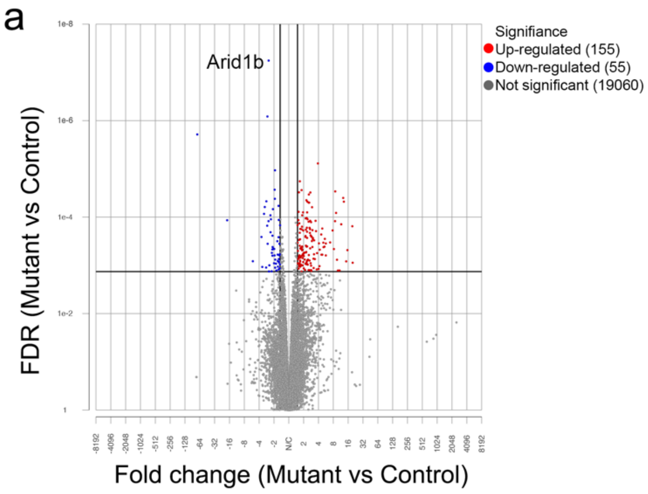

b

Top 20 changed genes

Gene ID	FDR step up (Mutant vs. Control)	Fold change (Mutant vs. Control)
Arid1b	0.00	-2.57
Adam23	0.01	-2.72
Hook1	0.04	-1.91
Wnt4	0.05	1.67
Myo16	0.05	-1.94
Afap1l2	0.05	1.82
Jun	0.05	1.60
Dsg1a	0.05	2.55
Atp1b1	0.05	2.59
Pkp1	0.05	2.54
Slc35d1	0.05	-1.62
Ptger3	0.05	-2.10
Tnfrsf19	0.05	1.58
Dsc3	0.05	1.94
Egfr	0.05	1.87
Chrdl1	0.05	-2.36
Dpep1	0.05	2.31
Irf6	0.05	2.20
Prss23	0.05	-1.60
Bcl11b	0.05	1.56

(FDR ≤0.1, Fold change <-1.5 or > 1.5, LSMean>10)

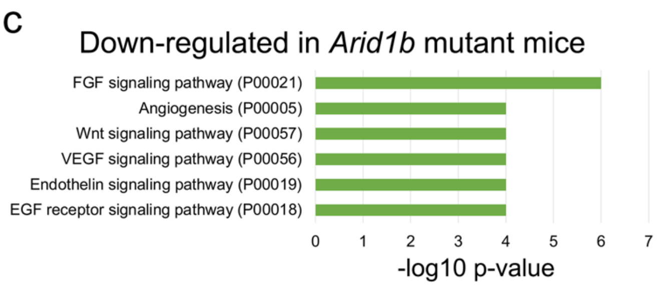

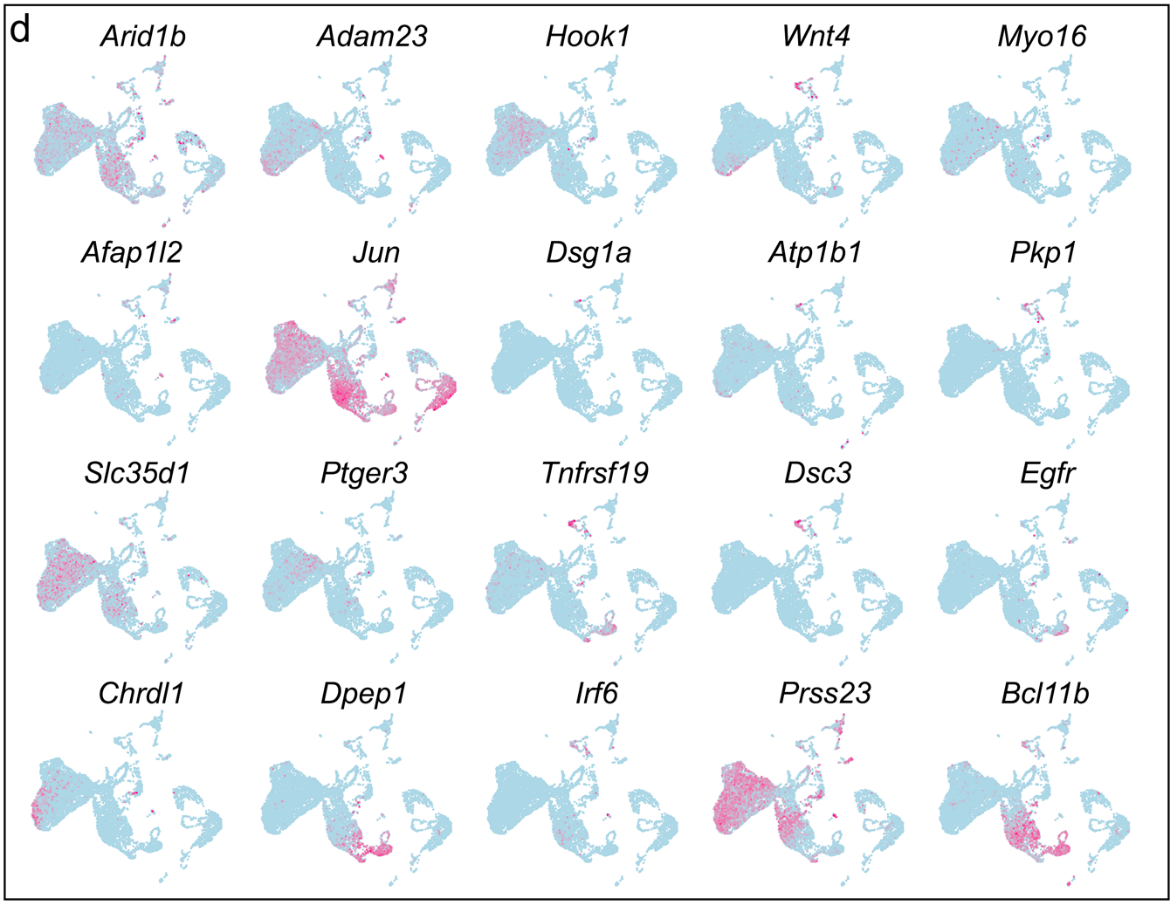

Supplementary Figure 5 Comprehensive scRNA-seq and bulk RNA-seq analysis between control and *Gli1-CreER;Arid1b$^{fl/fl}$* mouse incisor samples.

(a) Volcano plot of the bulk RNA-seq data from control and *Gli1-CreER;Arid1b$^{fl/fl}$* mouse incisors.

(b) Top 20 differentially expressed genes following the loss of *Arid1b* from the bulk RNA-seq analysis.

(c) Top signaling pathways identified by GO analysis using downregulated genes identified from bulk RNA-seq analysis.

(d) FeaturePlot of the top 20 differentially expressed genes using the scRNA-seq data from the control.

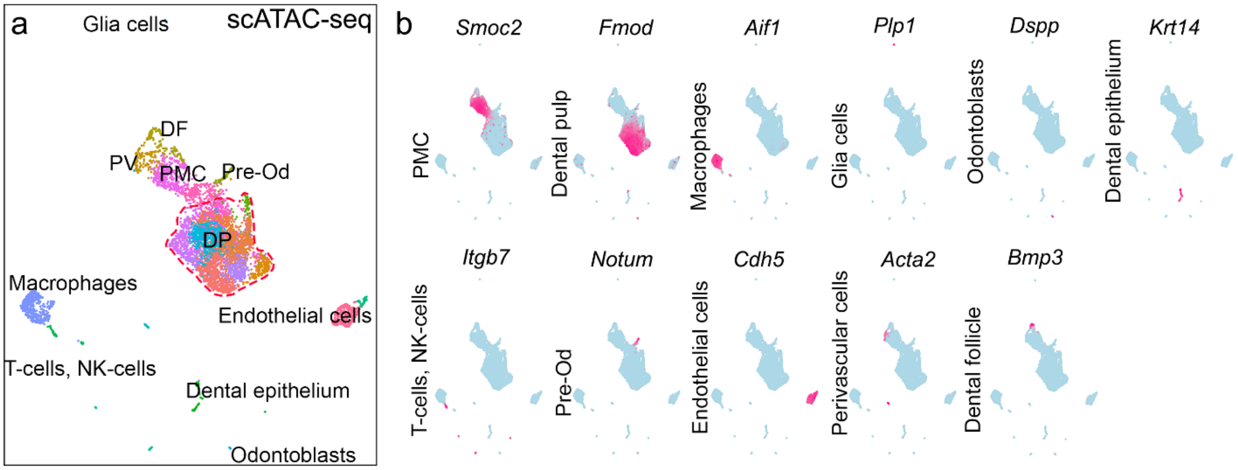

Supplementary Figure 6 scATAC-seq profiles of control and *Gli1-CreER;Arid1b$^{fl/fl}$* mouse incisor samples

(a) UMAP plot of all cell types in the mouse incisor and its surrounding tissue. DP, dental pulp; PMC, proximal mesenchymal cell; DF, dental follicle; Pre-Od, pre-odontoblast; PV, perivascular cell.

(b) FeaturePlot of marker genes in the mouse incisor and its surrounding tissue. Cell types were annotated based on the expression of cell type-specific marker genes.

a

BMP signaling related genes

Gene ID	FDR step up (Mutant vs Control)	Fold change (Mutant vs Control)
Bmp1	0.69	-1.16
Bmp2	0.93	-1.02
Bmp4	0.96	1.01
Bmp5	0.79	-1.09
Bmp6	0.85	-1.05
Bmp7	0.59	-1.09
Bmpr1a	0.73	1.07
Bmpr2	0.93	-1.02

(LSMean>10)

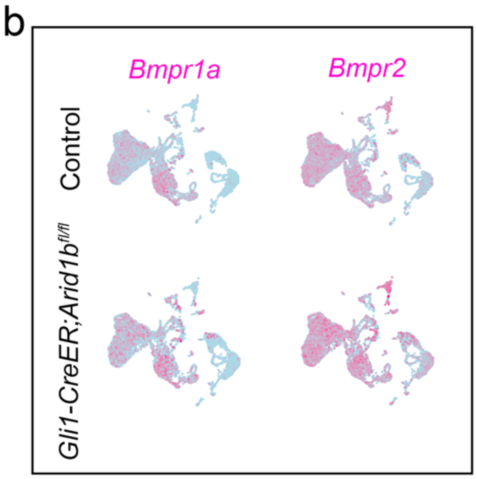

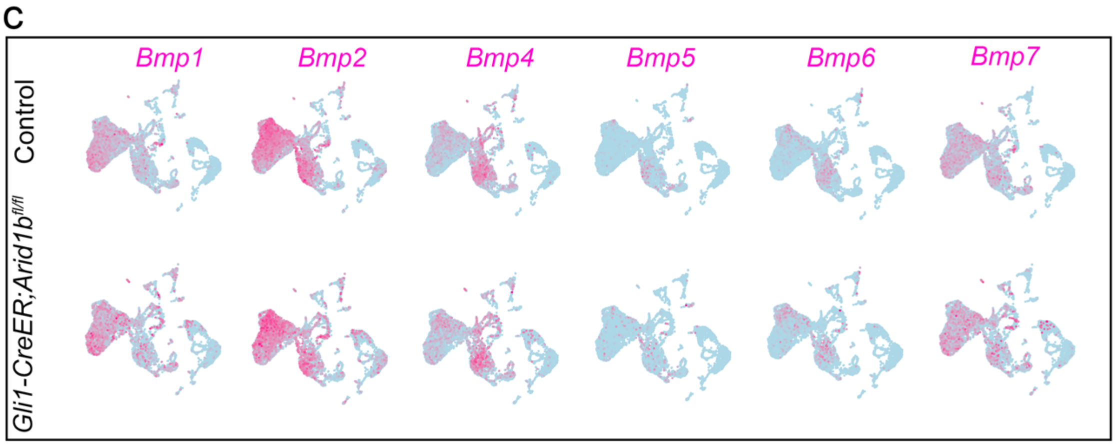

Supplementary Figure 7 BMP signaling is not significantly changed following the loss of *Arid1b* in the mouse incisor.

(a) Comparison of expression levels of BMP signaling ligands and receptors which are mainly expressed in the dental mesenchyme.

(b-c) FeaturePlot of receptors (b) and ligands (c) in BMP signaling pathway from control and *Gli1-CreER;Arid1b*$^{fl/fl}$ scRNA-seq data.

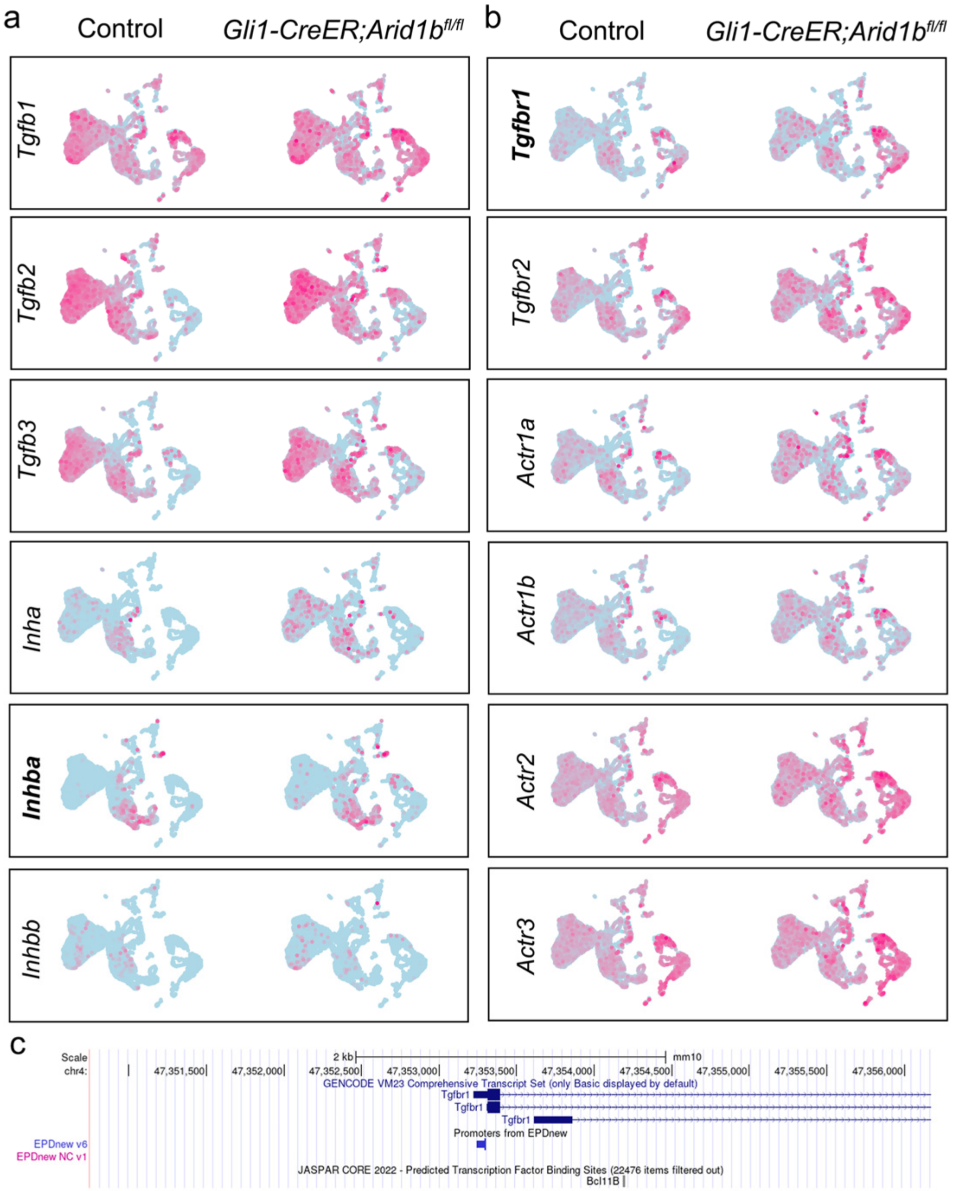

Supplementary Figure 8 *Inhba* and *Tgfbr1* are significantly upregulated following the loss of *Arid1b* in the mouse incisor.

(a-b) FeaturePlot of ligands (a) and receptors (b) in TGF-β signaling and Activin signaling pathways from control and *Gli1-CreER;Arid1b*$^{fl/fl}$ scRNA-seq data.

(c) UCSC binding prediction of BCL11B binding motif in the intron region of *Tgfbr1*.

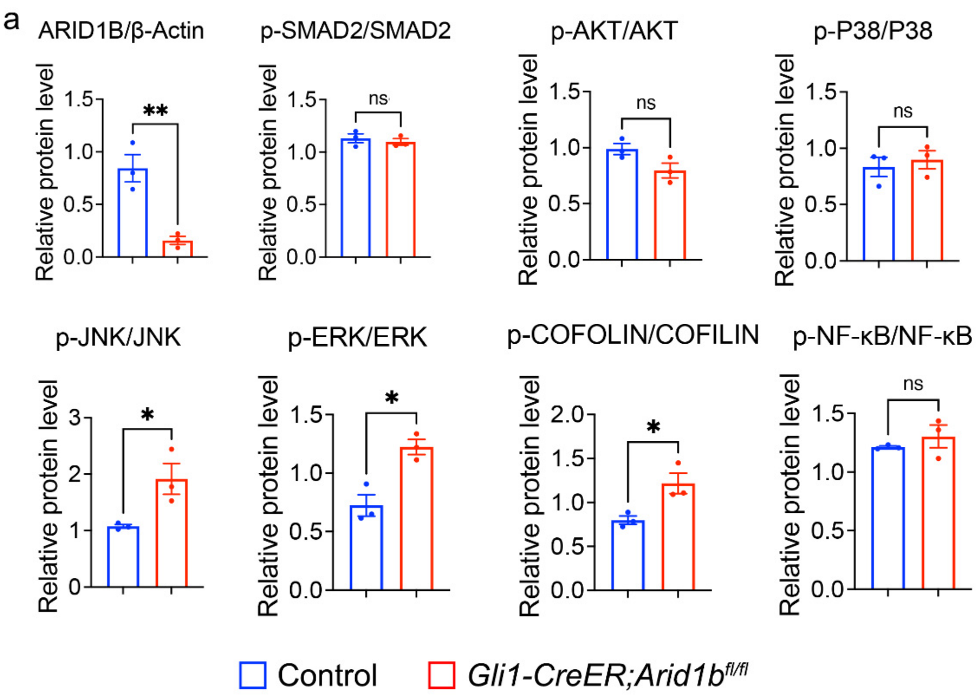

Supplementary Figure 9 Quantification of relative protein levels in main Figure 6a. Data are mean±SEM, n=3, unpaired two-tailed Student's t-test, ARID1B/β-Actin: p=0.0072; p-JNK/JNK: p=0.0378; p-ERK/ERK: p=0.0113; p-COFILIN/COFILIN: p=0.0295.

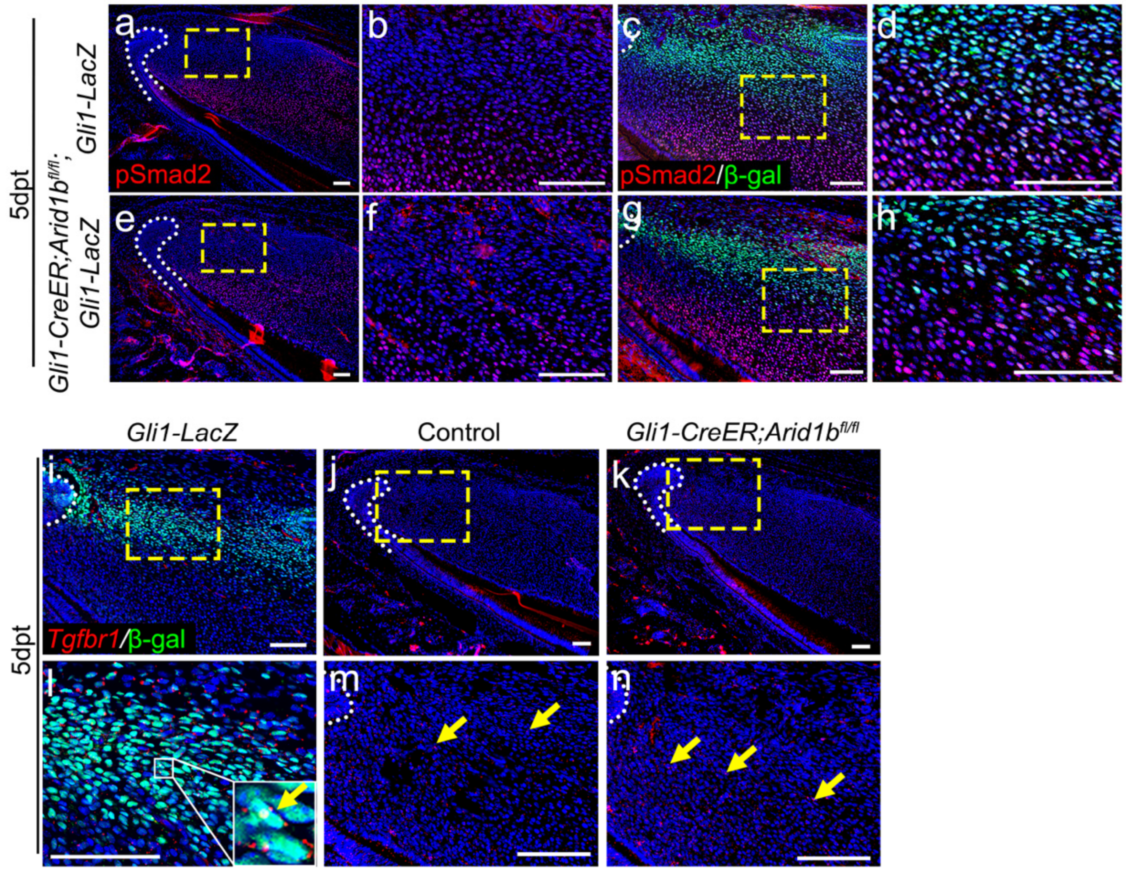

Supplementary Figure 10 Smad-dependent TGF-β/Activin signaling is activated in dental pulp cells adjacent to MSCs.

(a-h) Immunostaining of p-SMAD2 (red) and β-gal (green) in *Gli1-LacZ* (a-d) and *Gli1-CreER;Arid1b$^{fl/fl}$;Gli1-LacZ* (e-h) mouse incisors. b, d, f, and h represent high-magnification images of the boxes in a, c, e, and g, respectively. White dotted lines outline the cervical loop.

(i-n) *in situ* hybridization of *Tgfbr1* (red) and immunostaining of β-gal (green) in the *Gli1-LacZ* (i, l) mouse incisor, and comparison of *Tgfbr1* expression in control (j, m) and *Gli1-CreER;Arid1b$^{fl/fl}$* (k, n) mouse incisors. l, m, and n represent high-magnification images of the boxes in i, j, and k, respectively. White dotted lines outline the cervical loop. Yellow arrows indicate positive signals.

All the images are representative of at least 3 biological replicates. Scale bars: 100 μm.

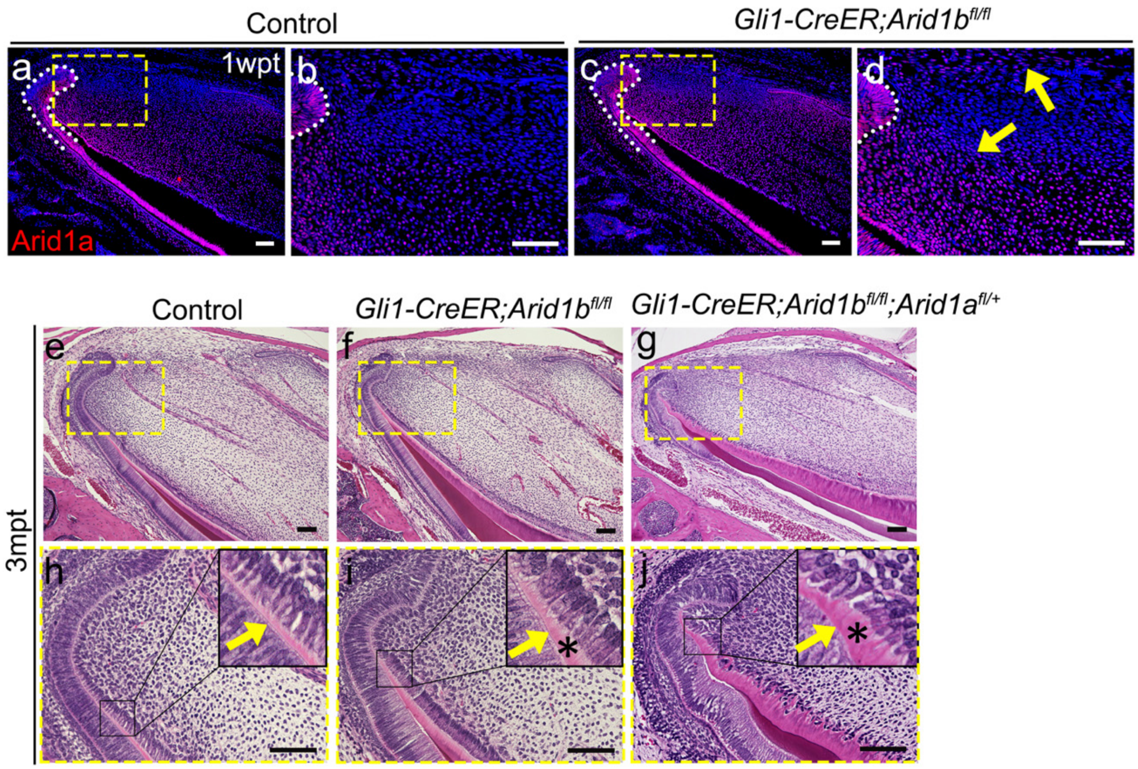

Supplementary Figure 11 ARID1A is essential in regulating mouse incisor tissue homeostasis in the *Gli1-CreER;Arid1b$^{fl/fl}$* mouse.

(a-d) Immunostaining of ARID1A in the incisors from control (a, b) and *Gli1-CreER;Arid1b$^{fl/fl}$* (c, d) mice 1-week post-tamoxifen induction. b and d represent the high-magnification images of the boxes in a and c, respectively. White dotted lines outline the cervical loop. Yellow arrows point to the positive cells.

(e-j) HE staining of incisors from control (e, h), *Gli1-CreER;Arid1b$^{fl/fl}$* (f, i), and *Gli1-CreER;Arid1b$^{fl/fl;}$Arid1a$^{fl/+}$* (g, j) mice at 3 months after tamoxifen induction. h, i, and j represent the high-magnification images of the dashed line boxes in e, f, and g. Yellow arrows indicate the initiation of odontoblast polarization. Black asterisks indicate stacked and distorted dentin.

All the images are representative of at least 3 biological replicates. Scale bars: 100 μm.
