# Supplementary figures and images for: ARID1B maintains mesenchymal stem cell quiescence via inhibition of BCL11B-mediated non-canonical Activin signaling
Source: Nat Commun. 2024 May 30;15:4614. doi: 10.1038/s41467-024-48285-2 (PMC11139927; doi:10.1038/s41467-024-48285-2)

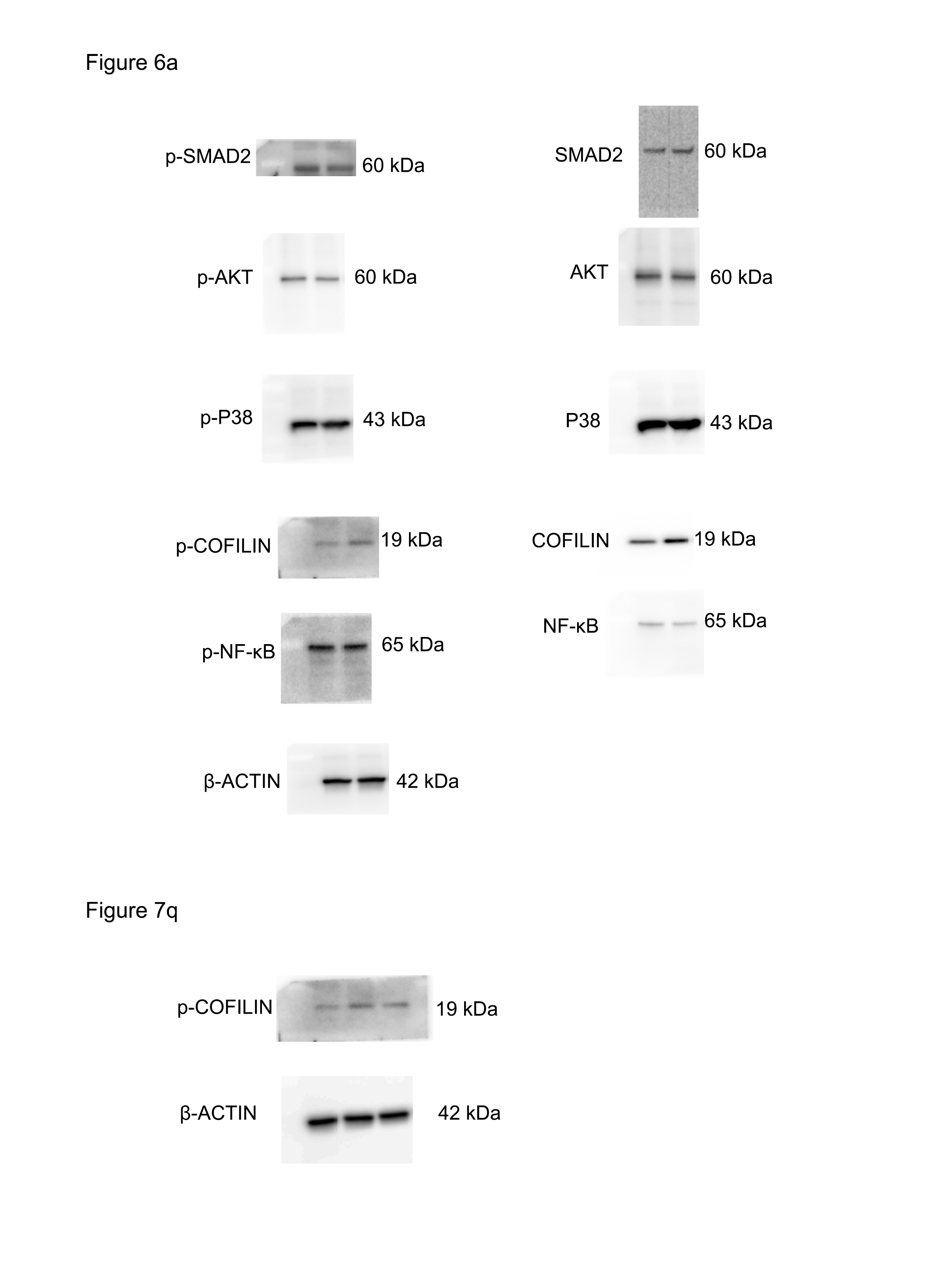

Supplement: Supplementary file 4 — Source Data [file 41467_2024_48285_MOESM4_ESM.zip › 447520_2_related_ms_8823113_slmh48.tif]
